# Implicit feedback policies for COVID-19: why “zero-COVID” policies remain elusive

**DOI:** 10.1038/s41598-023-29542-8

**Published:** 2023-02-23

**Authors:** Ali Jadbabaie, Arnab Sarker, Devavrat Shah

**Affiliations:** 1 Institute for Data, Systems, and Society, MIT, Cambridge, MA USA; 2 Department of Civil and Environmental Engineering, MIT, Cambridge, MA USA; 3 Department of Electrical Engineering and Computer Science, MIT, Cambridge, MA USA

**Keywords:** Control theory, Applied mathematics, Computational science

## Abstract

Successful epidemic modeling requires understanding the implicit feedback control strategies used by populations to modulate the spread of contagion. While such strategies can be replicated with intricate modeling assumptions, here we propose a simple model where infection dynamics are described by a three parameter feedback policy. Rather than model individuals as directly controlling the contact rate which governs the spread of disease, we model them as controlling the contact rate’s derivative, resulting in a dynamic rather than kinematic model. The feedback policy used by populations across the United States which best fits observations is proportional-derivative control, where learned parameters strongly correlate with observed interventions (e.g., vaccination rates and mobility restrictions). However, this results in a non-zero “steady-state” of case counts, implying current mitigation strategies cannot eradicate COVID-19. Hence, we suggest making implicit policies a function of cumulative cases, resulting in proportional-integral-derivative control with higher potential to eliminate COVID-19.

## Introduction

Determining the optimal policy for regulating an epidemic requires assessing complex infection dynamics across heterogeneous populations. Due to this complexity, simplified models of epidemic spread are often used in order to develop guidelines and understand the impact of policies on the level of infection. Throughout the COVID-19 pandemic, a wide range of models have been implemented in order to provide forecasts for important time series related to the pandemic as well as estimate causal effects of various policies^1–6^. Such approaches have been developed since as early as the 19th century, and have been successfully applied to forecast epidemics such as seasonal influenza, smallpox, and H1N1^7,8^.

However, a key distinction between the COVID-19 pandemic and the spread of infectious disease in the past is the extent to which populations have reacted to limit the spread of the virus. As cases have surged, government officials have instituted lockdowns and mask mandates, individuals have changed their behavior at an unprecedented scale, and the public has received access to mass vaccination. This endogenous response to the magnitude of the pandemic is rarely reflected by models developed for previous epidemics, but is critical in developing a robust response to COVID-19^9,10^. Even of the models used for forecasting COVID-19 fatalities, only one explicitly includes an assumption that policy changes as the state of the pandemic worsens^4^. That is not to say that other models do not account for behavior. Rather, the remaining predictive models which do account for behavior treat human intervention as an exogenous variable, observed for example through mobility data and the presence of government mandates and vaccinations.

In this work, we identify a parsimonious model for epidemics where the response to the pandemic is encoded as a feedback policy which depends on the number of cases observed so far. We assess the validity of the model by fitting it to empirical data on COVID-19 case counts, and find that the model fits data well both in and out of sample, despite only having three parameters. To develop a behavioral interpretation of the parameters of the model, we then compare learned parameters to explicit policy actions taken during the COVID-19 pandemic such as mobility restrictions and vaccination rates, which correlate well with the implicit parameters of the feedback law that encode the endogenous response of the population. Finally, we consider the long-term implications of the model and find that there is no currently implemented implicit policy within the global population that fully eliminates COVID-19. As such, we propose modifications to the implicit control which our model suggests would have a greater chance of achieving zero weekly COVID-19 cases.

### Model description

We begin by considering a general model of the growth phase of an epidemic with a time-varying growth rate, which we show contains several common epidemic models as special cases. We let *I*(*t*) represent the number of infections in a population at time *t*, where *t* is a discrete time index, and use the convention $I(0) = 1$. Generically, any epidemic process with time-varying growth rate can be written in the following form:

1 $$\begin{aligned} I(t+1) = I(t) \times {\mathcal {R}}(t) \times \eta (t) . \end{aligned}$$

Here, ${\mathcal {R}}(t)$ represents a generic time-varying parameter which indicates the number of infections that stem from each infected individual, and $\eta (t)$ represents noise in the process, which we assume to come from a log-normal distribution with parameters $\mu = 0$ and $\sigma ^2$. This choice of distribution for the noise is fundamental to the branching process literature that inspires this type of model and is a natural assumption in this setting^11^.

In this work, we build upon the aforementioned model and assume individuals control the growth rate using a time-varying parameter $\rho (t) \ge 0$ which represents the proportion of existing ties that each infected individual will add or remove at time *t*. Mathematically, we assume ${\mathcal {R}}(t) = {\mathcal {R}}(0) \times \prod _{k = 1}^{t-1} \rho (k)$, where ${\mathcal {R}}(0)$ is taken to be a constant value that represents the initial rate of infection. Hence, the open-loop dynamics of the system considered in this work take the form

2 $$\begin{aligned} I(t+1) = I(t) \times {\mathcal {R}}(0) \times \prod _{k = 1}^{t-1} \rho (k) \times \eta (t) . \end{aligned}$$

Although equating $\rho (t) = {\mathcal {R}}(t+1) / {\mathcal {R}}(t)$ reveals that the models in Eqs. (1) and (2) are equivalent, we note that the parameterization of the model in terms of $\rho (t)$ represents a subtle but important distinction from models proposed in recent literature: rather than assume the contact rate is a direct function of a population’s interventions, here we make the assumption that individuals are instead controlling the *rate* at which they increase or decrease interactions with one another, resulting in a dynamic as opposed to a kinematic model. In terms of a mechanical analogy to a moving car, rather than assuming that individuals dictate control of position through a choice of velocity, our model assumes that individuals control their position through acceleration, i.e. by pressing on the gas or the brakes. This parameterization allows us to model the reaction of the population in a novel way by allowing $\rho (t)$ to be a function of previous case counts, as will be shown in Eq. (4).

*Connections to existing epidemic models* Because the time series of control parameters $\rho (t)$ is not specified in Eq. (2), the model is considered open-loop, and we note that natural selections of $\rho (t)$ lead to common epidemic models in the literature. In particular, if $\rho (t) = 1$ for all *t*, then the model leads to *exponential growth* in case counts, which is a commonality across the initial stages of many epidemic models^12^.

If $\rho (t) = {\bar{\rho }}$ for all *t*, where ${\bar{\rho }} < 1$, then Eq. (2) recovers the form of the Gaussian curve noted in *Farr’s law*, which is a common non-mechanistic approach to prediction of an epidemic^7^. This model underlies many common data-driven approaches to epidemic modeling and forecasting, cf.^6,13^.

Moreover, a detailed connection can be made to the standard SIR model. A specific time-varying choice of $\rho (t)$ can recreate the traditional *Susceptible-Infected-Recovered (SIR)* model as well as its time-varying extensions^14^. For simplicity of exposition, here we consider the most basic SIR model of epidemic spread. One can make similar comparisons to variants of the model such as SEIR, SIR with vaccination, or other compartmental models^12^. The SIR model and relevant variants have been used extensively throughout the COVID-19 pandemic, for both forecasting and inference of population dynamics (a non-exhaustive list includes^1–3,15–17^). In discrete time, the SIR model consists of the following set of dynamic equations^18^:

$$\begin{aligned} S(t+1) - S(t)&= - \frac{\beta }{N} S(t) I(t) \\ I(t+1) - I(t)&= \frac{\beta }{N} S(t) I(t) - \gamma I(t)\\ R(t+1) - R(t)&= \gamma I(t) \,. \end{aligned}$$

In the model, *N* represents population size, and *S*(*t*), *I*(*t*) and *R*(*t*) represent the parts of the population that are susceptible, infected, and recovered from infection, respectively. In this simplified model, it is assumed that for all *t*, $S(t) + I(t) + R(t) = N$, and we use the convention that at $t = 0$ we have $I(0) = 1, R(0) = 0$, and $S(0) = N - 1$. The parameters $\beta$ and $\gamma$ modulate the dynamics of the system, where $\beta$ measures the average number of contacts each individual in the population has with others, and $1/\gamma$ represents the average amount of time that an infectious individual is able to infect others. Such parameters are often allowed to be time-varying, which ultimately results in infection dynamics of the form

3 $$\begin{aligned} I(t+1) = I(t) \times \left[ 1 - \gamma (t) + \frac{\beta (t)}{N} S(t) \right] . \end{aligned}$$

For any choice of time-varying $\gamma (t)$ and $\beta (t)$, these dynamics are a special case of the model Eq. (2), as we note in the following proposition, proved in Online Appendix A.

#### Proposition 1

There exists a parameterization of the open-loop model in Eq. (2) such that its dynamics are equivalent to the time-varying SIR model in Eq. (3).

The connection to the SIR model reveals that the model in Eq. (2) provides a novel way to represent how individuals react in a pandemic. Specifically, we see that the open-loop model in Eq. (2) does not explicitly assume that individuals in a population are adjusting their contact rates or recovery rates. The model assumes that people are unaware of the cardinal values that govern the physics of infection dynamics, given by a particular value of ${\mathcal {R}}(t)$, and instead control the relative proportion of ties they add or remove from their network, which would be equivalent to ${\mathcal {R}}(t) / {\mathcal {R}}(t-1)$.

*A new model of implicit feedback* While there are many possible ways in which feedback can be incorporated into the model in Eq. (2), we find that observed COVID-19 case counts are consistent with a particular type of feedback control law known as a proportional-derivative (PD) controller. Specifically, the data is consistent with a control parameter $\rho (t)$ which takes the following form:

4 $$\begin{aligned} \log \rho (t)&= \beta _1 \times \log I(t)~ \nonumber \\&\quad +\beta _2 \times (\log I(t) - \log I(t-1) - \log {\mathcal {R}}(0)) + \beta _3 \,, \end{aligned}$$

where $\log I(t)$ represents the proportional term (P) and $\log I(t) - \log I(t-1) - \log {\mathcal {R}}(0)$ represents the derivative term (D). This particular form for the control input is selected for the following reasons. First, the model appears to have the best performance compared to other feedback models (see “Comparisons to other feedback models” section). Second, the parameterization of the control input is inspired by a long history of research in control engineering which studies the behaviors of proportional (P), proportional-derivative (PD), and proportional-integral-derivative (PID) control. This allows us to compare the model in Eq. (4) to other well-known feedback policies. Moreover, the model is parsimonious in that it only relies on three parameters and these three parameters have a simple behavioral interpretation.

Behaviorally, the first term in the sum of Eq. (4) implies that individuals in a population observe and react to case counts through $\log I(t)$. If $\beta _1 < 0$, which is the case in observed data, then larger magnitude of cases result in more control actions, for example through increased social distancing or mask adoption. Similarly, individuals react to the recent change in cases relative to the reproductive rate, and when $\beta _2 < 0$, which is also the case in observed data, then individuals increase the control action if cases are rising quickly. Finally, $\beta _3$ represents a constant term which indicates an inherent level of reaction by the population. While these parameters need not be related, we note empirically that they tend to be correlated with one another.

It is worth noting that if $\beta _1 = \beta _2 = \beta _3 = 0$, then $\rho (k) = 1$ in this uncontrolled case and we recover a simple model of exponential growth. Moreover, as discussed in Online Appendix A, this assumption can be viewed as creating a specific time-varying structure for the time-varying SIR model in Eq. (3). Using state feedback allows us to write the model into the single closed loop dynamical system as follows.

Let ${\textbf{X}}(t) \in {\mathbb {R}}^3$ represent the state of a dynamical system where $X_1(t) = \log I(t-1)$, $X_2(t) = \log I(t)$, and $X_3(t)$ represents the cumulative control taken so far, i.e. $X_3(t) = \sum _{k = 1}^{t-1} \log \rho (k)$. The closed loop dynamics of the system Eq. (2) under the control policy Eq. (4) can be shown algebraically to be summarized by the following affine dynamical system:

5 $$\begin{aligned} {\textbf{X}}(t+1)&= {\textbf{Q}} {\textbf{X}}(t) + {\textbf{c}} + \varvec{\eta }(t) \,. \end{aligned}$$

Here,

$$\begin{aligned} {\textbf{Q}} = \begin{bmatrix} 0 &{} 1 &{} 0 \\ 0 &{} 1 &{} 1 \\ - \beta _2 &{} \beta _1 + \beta _2 &{} 1 \end{bmatrix} \,, ~\text {and}~{\textbf{c}} = \begin{bmatrix} 0\\ \log {\mathcal {R}}(0) \\ -\beta _2 \log {\mathcal {R}}(0) + \beta _3 \end{bmatrix} \,. \end{aligned}$$

The details of this derivation are presented in Online Appendix B. Given the assumption that $\eta (t)$ in Eq. (2) is log-normal, we see that $\varvec{\eta }(t)$ can be modeled as unobserved Gaussian noise with mean 0 and variance $\sigma ^2$. This closed loop model is extremely simple as it is governed by only three parameters, and can be efficiently learned from data^19^. As our results indicate, the model with the PD controller fits the data surprisingly well, which suggests that it succinctly describes existing behavior throughout the pandemic, and the model has the critical implication that steady state infections are non-zero.

### Summary of results

With the above model, in comparing to existing data we see that there is a satisfactory fit to observed data and that the parameters of the implicit feedback correlate with observed policies. Moreover, in our analysis, we find that a major consequence of this model is that weekly cases are never eradicated; rather, for most observed learned parameters, weekly cases will stabilize at some non-zero level. This has non-trivial policy implications– in order to eradicate COVID-19 sooner rather than later, populations need to introduce another policy intervention. For example, by reacting to *total* case counts since the beginning of the pandemic rather than the case counts in the last two weeks.

*Fit to data* To assess the validity of the proposed model, we fit Eq. (5) to observed case counts during the COVID-19 pandemic. Our results indicate the model fits to empirical data surprisingly well both in and out of sample, despite having only 3 learned parameters, which suggests that the model captures the essence of population dynamics in reaction to COVID-19 case counts. As a means of comparison, we assess the performance of our model against the ensemble method used by the CDC, which aggregates predictions from at least 30 state of the art forecasting models each week to provide predictions of COVID-19 case counts^5^. When comparing the simple 3 parameter model to ensemble forecasts at the state level, we find that the prediction performance is comparable for 1, 3, and 4 week ahead forecasts. Moreover, our results suggest the model in Eq. (5) becomes competitive in longer term forecasting, which is consistent with prior results on the use of feedback in forecasting^17^. We also show that other similar feedback policies do not fit as well to the data as the simple policy suggested in Eq. (4). This, combined with the comparison to observed policies noted below, supports the claim that populations implicitly perform PD control.

*Comparison between implicit and explicit policies* In regressing parameters of the implicit control against explicit policies taken by different populations across the globe, we find significant correlations between the $\beta _1$ and $\beta _2$ parameters when compared to mobility data as well as levels of natural immunity measured by the percentage of the population which has had a confirmed positive COVID-19 test. This relationship between parameters of the model and observed policies taken by populations reinforces the validity of the model and also suggests ways in which the effect of the pandemic may be mitigated which are consistent with the prevailing public health guidance in the United States.

*Difficulty of eradication and proposed modifications* We show that there is no selection of $\beta _1,$ $\beta _2$, and $\beta _3$ that has been implemented by states which would result in zero weekly cases. As a result, in order to fully eliminate the COVID-19 pandemic, populations must alter their implicit strategies and deviate from the dynamical model in Eq. (5). This result is consistent with previous results on the fragility of non-pharmaceutical interventions, which suggests that even small measurement errors in the timing of a strategy can produce large increases in case counts^16^. Our results expand on this prior work by incorporating recent data, suggesting that even pharmaceutical interventions such as vaccinations are currently insufficient to eradicate new COVID-19 cases. Further, the results presented here are empirical in nature, and highlight properties of implemented policies.

We conclude by presenting an example of a policy which does result in the eradication of COVID-19 cases in theory, namely a proportional-integral-derivative (PID) control. Although our data suggests that no state or country currently implements an implicit policy with PID control, we provide steps which suggest a time-varying policy that would replicate PID control. Our results suggest that by increasing the intensity of certain interventions such as vaccination rates by an order of magnitude, COVID-19 cases can be eradicated.

## Data

The data used in order to learn the parameters of the model is taken from Johns Hopkins^20^. In preprocessing the data, we assume each time index *t* represents a full week to remove weekly seasonality. The dataset provides time series of case data in each state of the United States, as well as for 110 countries, which we aggregate weekly to fit the model in Eq. (5). We also use this data to compute estimates of naturalized immunity, by taking the ratio of confirmed positive tests and each region’s population to get a percentage of individuals who have had COVID-19.

In order to compare the learned parameters to observed policies, we also use data from Google^21^, which compares aggregate mobility trends across individual states and countries compared to pre-pandemic levels.

## Methods

To learn the parameters of the implicit control, we focus on identifying the parameters $\beta _1$, $\beta _2$, and $\beta _3$ from Eq. (4). To do so, we first infer each $\log \rho (t)$ from future time steps, as Eq. (2) yields:

$$\begin{aligned} \log \rho (t) = \log I(t+1) - \log I(t) - \sum _{k = 1}^{t-1} \log \rho _k - \log {\mathcal {R}}(0) - \log \eta (t) . \end{aligned}$$

Hence, because $\log \eta (t)$ is mean-zero, we estimate

6 $$\begin{aligned} \log \rho _0&= \log I(t+1) - \log I(t) - \log {\mathcal {R}}(0) \nonumber \\ \log \rho (t)&= \log I(t+1) - \log I(t) - \sum _{k = 1}^{t-1} \log \rho (k) - \log {\mathcal {R}}(0) ~~ (t \ge 1) \,. \end{aligned}$$

We estimate ${\mathcal {R}}(0)$ to be equal to 2.5^22^, and hence we are able to infer the values of the dependent variable $\log \rho (t)$.

We then perform a least-squares regression to determine the coefficients $\beta _1$, $\beta _2$, and $\beta _3$ in Eq. (4), where the exogenous variables are computed from time series data ($\log I(t)$, $\log I(t) - \log I(t-1) - \log {\mathcal {R}}(0)$, and a constant term) and the endogenous variable is given by Eq. (6) above, which we consider a measure of the true control taken by a population. This approach of using a least-squares regressions to learn the parameters of a dynamical system is common in the literature, and many theoretical results exist showing the consistency and non-asymptotic reliability of such methods^19^.

We also provide statistical evidence which shows that the learned $\beta _1$ and $\beta _2$ parameters above strongly correlate with policies taken by individuals during the pandemic (See Tables S1, S2, and Fig. S5). To get an estimate of policies taken during the pandemic, we process the Google mobility data, which consists of six mobility measures given as time series for each geographical region, and take the first two principal components of the data, such that for each region we get two time series. We then average this time series for each region over the time period that the policies are learned, giving two regressors for each region. Finally, we compute the correlation between the $\beta _1$ and $\beta _2$ parameter and each mobility metric. We then repeat the same procedure replacing the mobility metrics with the normalized number of cumulative cases in each region up to the end of the training period, which we use as a proxy for natural immunity.

## Results

### Fit to observed data

**Figure 1:**
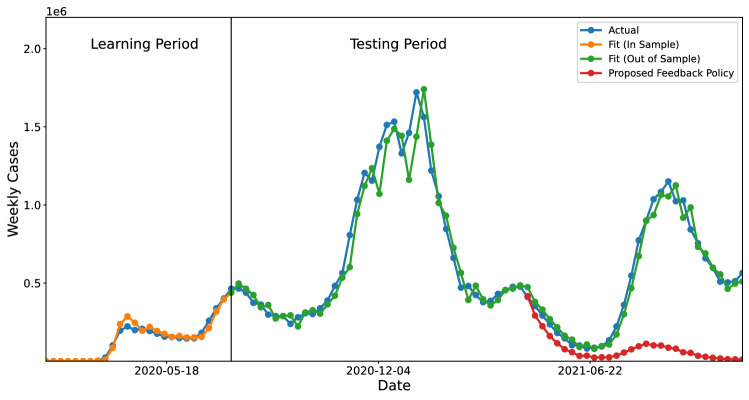
US Case counts as modeled by the dynamical system in Eq. (5). We learn the parameters of the control $\beta _1$ and $\beta _2$ on the first 30 weeks of data, and then they are held constant for the remainder of the data. One week ahead predictions using this method are shown in orange (in-sample) and green (out of sample), and the true data is shown in blue. The red line represents a transition to a separate proposed policy which incorporates an integral feedback term, as discussed in Rethinking Control for Future Epidemics.

To validate the model, we fit it to observed case counts throughout the COVID-19 pandemic to determine if it captures population dynamics. As shown in Fig. 1, the closed-loop model Eq. (5) fits the data well on United States national level case counts, and we additionally fit the model to data across various times, regions, and forecast targets in order to validate the model. For example, we find that the model tracks case counts across all 50 states well (Supplementary Information, Fig. S1). As a quantitative baseline, we consider state of the art ensemble forecasts which aggregate predictions from over 30 state of the art prediction models^5^, and find that the fit of the closed loop model is often comparable to the state of the art forecasts (Supplementary Information, Table S1). It is worth noting that the ensemble forecasts are used solely as a baseline to validate that the model in Eq. (5) fits well to the data. In practice, due to delays in the data collection, the observed values of *I*(*t*) at time *t* are often underestimated, resulting in a small drop in predictive performance when only data available at each particular time is used (Supplementary Information, Fig. S6 ). Although such data fidelity issues can be accounted for, we note that the purpose of this work is to highlight the behavioral implications of this model as opposed to its predictive value.

In comparing the closed-loop model Eq. (5) to ensemble predictions for forecast targets ranging from 1 to 4 weeks ahead of the forecasting date, we find that the implicit control approach has comparable $R^2$ scores to the ensemble approach for $k = 1, 3,$ and 4, as shown in Fig. S2. Taking the United States national case counts as an example, as illustrated in Fig. 1, we find that for the in-sample time series, the model fits the data with an $R^2$ value of 0.966, and for the out of sample time series, the $R^2$ value is 0.949. In comparison, the 1 week ahead forecasts of state of the art ensemble predictions over the same period is 0.942^5^.

**Figure 2:**
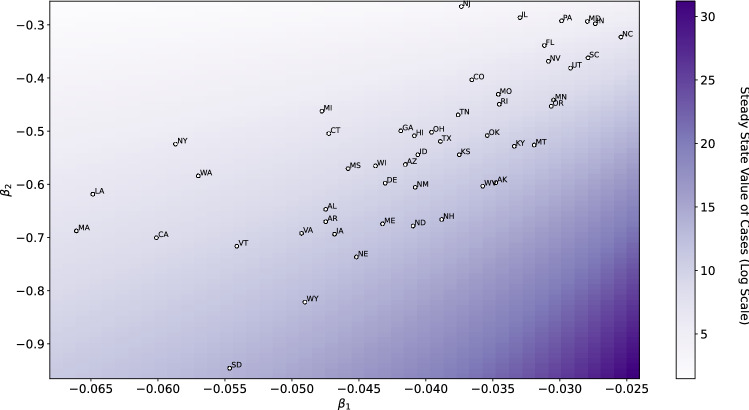
Coefficients of the implicit control used by the 50 US states. We find that states with low magnitude of $\beta _2$ and high magnitude of $\beta _1$ have a higher magnitude of steady state cases, highlighting a need for adjusted implicit policies in states such as South Dakota, Wyoming, and Nebraska.

The learned $\beta _1, \beta _2$, and $\beta _3$ parameters are heterogenous between different geographic regions, and we find that different parameters of implicit control result in different implications for the pandemic. The 50 states in the US provide insight into the different values of $\beta _1$ and $\beta _2$ and their importance in understanding the different forms of control that are being implicitly used by different states throughout the pandemic. The learned $\beta _1$ and $\beta _2$ parameters from each of the 50 states are shown in Fig. 2, and it is clear that there is heterogeneity in the learned values.

**Figure 3:**
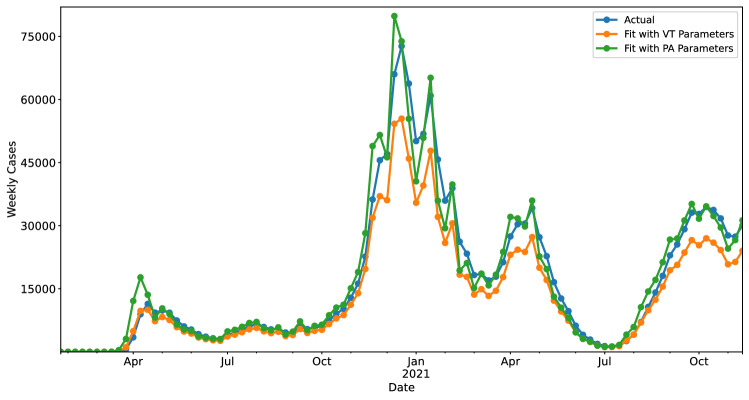
Applying learned parameters across different states. When applying the parameters learned from Vermont (VT) data to fit the data from Pennsylvania (PA), we find that the model predictions are drastically different. This suggests that variations in the control parameters $\beta _1$, $\beta _2$, and $\beta _3$ are meaningful.

The differences in these learned values are significant as far as their effects on prediction, as we can not simply take one state’s parameters and use this information to predict for a different state (Fig. 3). While there are clusters of states with similar parameters, it is clear that the specific values of parameters is still important in terms of generating valid predictions and explaining behavior.

**Figure 4:**
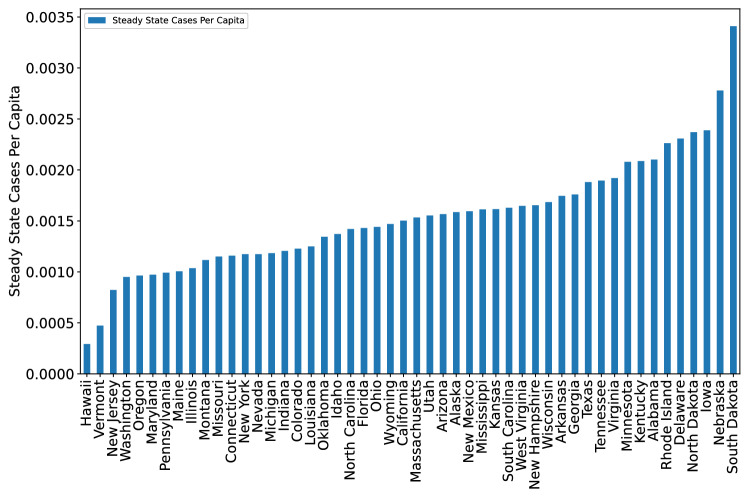
Computed steady state case counts per capita for each state, based on the learned parameters of the implicit control policy. These values have a 0.564 correlation with the actual mean weekly case counts per capita in each state $(p < 10^{-4})$, supporting the validity of the model Eq. (5) and suggesting particular geographic regions to focus on adjusting implicit control.

The learned $\beta _1$, $\beta _2$, and $\beta _3$ parameters can also be used in order to understand the heterogeneity in each state’s implicit control policies. When the matrix ${\textbf{Q}}$ is Hurwitz (has spectral radius at most 1) in Eq. (5), we can compute the steady state number of cases $\lim _{t \rightarrow \infty } X_2(t)$. We report the per capita results in Fig. 4. This steady state value provides insight on how different states handled the pandemic in terms of the ability to efficiently bring case counts towards 0. In particular, larger steady state values seem to suggest relaxed policies in controlling pandemic.

It is worth noting that the model captures a compounded impact of government policies, population behavior and other circumstances (e.g. weather, industry, etc.). However, it is a definitive way to “evaluate” the compounded effect across states which may be of interest in its own right.

### Relationship to observed policies

Our results indicate that both $\beta _1$ and $\beta _2$ in Eq. (4) correlate with observed policies taken throughout the pandemic. In comparing the learned parameters of all 50 states as well as 110 countries to observed mobility patterns, we find statistically significant correlations (Supplementary Information, Table S2). Moreover, we also see significant correlations with respect to the proportion of the population that has become immune during the training period. This suggests a relationship between learned parameters and the proportion of the population which is no longer susceptible to the disease. In fact, a similar relationship can be shown between $\beta _1$ and vaccination rates across the United States; however, the statistical evidence only suggests a correlation as the parameters are learned from the initial stages of the pandemic, so a causal relationship can not be made (Supplementary Information, Table S3).

### Comparisons to other feedback models

While the model fits to empirical data suggest that the model is consistent with observations, and the parameters do correlate with observed policies, such results cannot definitively prove that the model assumptions are representative of reality. Here, we compare our model to other plausible hypotheses in order to understand the value and limitations of this simple approach.

Because the results until now have provided one week ahead forecasts, and hence are dependent on most recent actual observations, it is also important to compare against a case in which these recent observations are estimated. In control theory, an observer is used for this task^23^. When implementing the system with an observer as opposed to true data on the US case counts, we find that our predictions are still very close to when the original data is used, with nearly equivalent $R^2$ values (Supplementary Information, Fig. S4).

**Figure 5:**
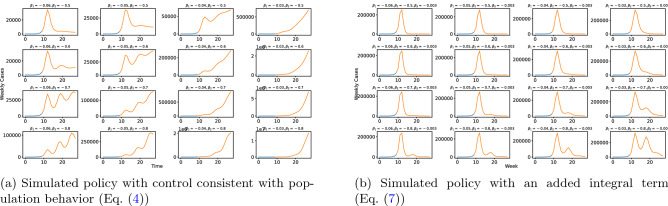
(**a**) Different choices of $\beta _1$ and $\beta _2$ for the system in Eq. (5). This simulated system begins with an initial exponential growth (blue) followed by case counts when the control policy enacted (orange). We find that as $\beta _1$ becomes closer to 0, the system becomes increasingly unstable and prone to exponential growth. As $\beta _2$ decreases away from 0 with fixed $\beta _1$, we see that the system becomes more oscillatory and likely to become unstable as well. Moreover, across all selection of parameters, even when the system is such that ${\textbf{Q}}$ is stable, we see that the steady state number of cases is non-zero. (**b**) Adding an integrator term $\beta _S \sum _{k=1}^t X(k)$ to the implementation of the control input $\log \rho _t$. The simulated system again begins with an initial exponential growth (blue) followed by case counts when the control policy enacted (orange). The integral term stabilizes the system and brings weekly case counts towards an steady state with no weekly cases, although in practice we find that no country has a statistically significant non-zero $\beta _S$ term in its control policy.

We also compare this model to a simple, one-dimensional Proportional-Integral-Derivative (PID) controller, since PID control is nearly ubiquitous in control applications^24^. In this comparison, we find that the PID controller requires more data in order to effectively learn appropriate parameters, and that it does not explain the data as well as the closed-loop system in Eq. (5) (Supplementary Information, Fig. S3). Hence, although PID control is omnipresent in engineering applications, it is not representative of the actual behavior observed throughout the pandemic (Figs. 4 and 5).

**Figure 6:**
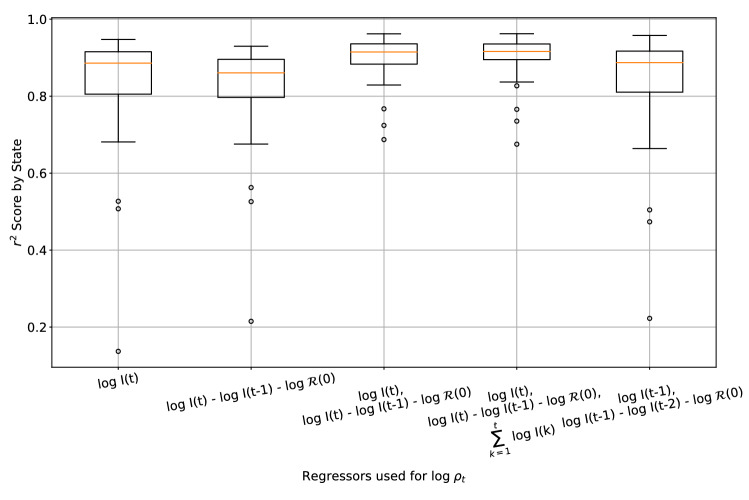
Boxplot of $R^2$ scores of the 50 US states when different regressors are used to determine $\log \rho _t$. We find that the choice of regressors in Eq. (4) provides the best fit to the data overall, suggesting that the three parameter control input fits sufficiently well across heterogenous regions. Moreover, including a regression term on $\sum _{k = 1}^t \log I(t)$, as is proposed in Eq. (7) does not result in an improved fit to the data, suggesting that this particular policy which would eradicate COVID-19 cases is not being implemented by populations.

Finally, we compare against several models for which different sets of regressors are used in estimating $\log \rho _t$, in order to understand the different implications of each approach (Fig. 6). Our results indicate that our proposed feedback law in Eq. (4) provides the best explanation of $\log \rho _t$ across the 50 states. It is also important to note that the use of $\sum _{k = 1}^t \log I(t)$ as a regressor for $\log \rho _t$ does not improve prediction performance, and that the coefficient of $\sum _{k = 1}^t \log I(t)$ is never significantly away from 0 across all 50 states and countries considered. This observation indicates at least one way in which policy changes can be modified to eradicate COVID-19 cases, and will be discussed further in Rethinking Control for Future Epidemics where we discuss the policy implications of our results.

### Properties of the implicit feedback control

The impact of the parameters which govern the implicit feedback control is summarized in Fig. 5a. Figure 5a indicates that there is a complex relationship between the values of $\beta _1$ and $\beta _2$ and the resulting case counts. Namely, increases in $\beta _2$ often result in oscillatory behavior, but still depend on the value of $\beta _1$. These plots reflect that this control strategy results in a constant but non-zero steady state of weekly cases. Stated differently, the this approach suggests that behavior eventually results in an instantaneous reproductive rate of 1. This reinforces that the system is sensitive to the selection of $\beta _1$ and $\beta _2$, and our results indicate that $\beta _1$ and $\beta _2$ can be learned from data and fit the data surprisingly well (Fig. 1).

### Rethinking control for future epidemics

Principles of control dictate that when Eq. (2) has a multiplicative factor of ${\mathcal {R}}(0) > 1$, the system generated when taking logarithms of Eq. (2) can be driven to 0 weekly cases using a feedback law which includes an integral term. That is, rather than the system dynamics being governed by the form of $\log \rho (t)$ described in Eq. (4), a suitable control would take the form

7 $$\begin{aligned} \log \rho (t)&= \beta _1 \times \log I(t)~ \nonumber \\&\quad +\beta _2 \times (\log I(t) - \log I(t-1) - \log {\mathcal {R}}(0)) ~\nonumber \\&\quad +\beta _3 + \beta _S \times \sum _{k = 1}^t \log I(t) \,, \end{aligned}$$

where $\sum _{k = 1}^t \log I(t)$ represents an integral feedback control. On synthetic data, the effect of this integrator term $\beta _S \sum _{k=1}^t \log I(t)$ is clear, as it drives weekly case counts to 0 (Fig. 5b). This additional term forces the weekly reaction of the community to the pandemic to go from being a proportional derivative (PD) control in Eq. (4) to a proportional-integral-derivative (PID) control, which provides the necessary integration of error which eventually results in a steady state of 0 weekly cases.

*Key policy implication* Ultimately, this suggests that the *cumulative* costs of the pandemic should be emphasized, even when the current state of the pandemic has a low number of cases. When the implemented control has little memory of the past, we can not expect cases to decay towards 0. The overall policy implication here is *implicit*, rather than explicit, in the sense that the current policy interventions being used, such as stay at home orders, mask mandates, and vaccination development, are still suggested by this model. Rather than implementing these policies as a function of recent cases, the model suggests they should be implemented and intensified according to cumulative case counts. Moreover, because the suggestion is implicit in behavior, the model also calls for individuals in a population to adopt a slightly different latent approach that reduces contacts as a function of cumulative case counts as opposed to recent ones.

**Figure 7:**
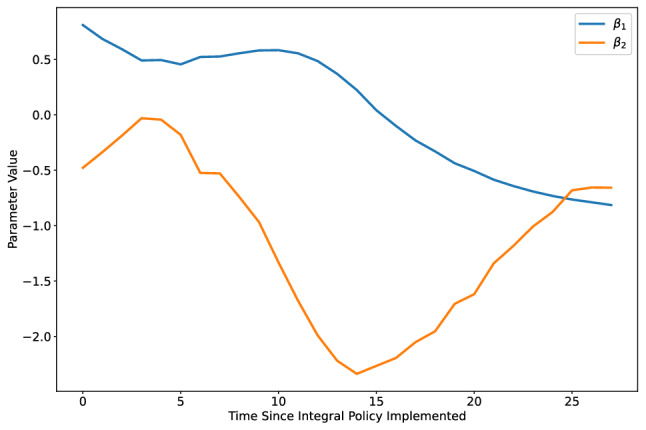
Example of recreating the integral feedback policy of the red line in Fig. 1 using time-varying $\beta _1$ and $\beta _2$ to replicate the effect of an integral feedback term. By steadily increasing vaccination adoption according to the blue line presented above, and using stay at home orders to replicate the orange line, policy makers can effectively replicate an integral feedback policy.

While there are many ways integral feedback can be implemented in practice, we highlight one possible policy which can be taken by adapting the existing policy in Eq. (4) using time-varying parameters. In Fig. 7, we show that the integral policy suggested Fig. 1 can be replicated by allowing $\beta _1$ and $\beta _2$ to be time-varying. Moreover, since we have shown that $\beta _1$ and $\beta _2$ relate linearly to observed policies such as vaccination rates and mobility above (Supplementary Information, Fig. S5), this suggests that using time-varying policies can recreate an integral feedback policy. Specifically, in the United States we find that $\beta _1$ relates collinearly with vaccination rates, and that $\beta _2$ related collinearly with percentage of time individuals spend at home. Using these policy levers in a way that is consistent with Fig. 7 would effectively replicate an integral feedback policy over time. Because the values of $\beta _1$ in Fig. 7 are approximately ten times larger in magnitude than those learned across the 50 states, the analysis suggest that any appropriate policy would require that interventions would need to be increased by an order of magnitude in order to eradicate COVID-19 cases.

## Discussion

We provide a dynamical model to represent the trajectory of cases in an epidemic, and show that COVID-19 cases are well explained by a control strategy which only depends on three parameters. The result is based on a robust estimation procedure, and all learned parameters are non-zero in a statistically significant sense.

The model takes an approach which is dynamic rather than kinematic. That is, we assume that individuals only control the rate at which they increase or decrease contacts with one another, as opposed to controlling the number of contacts with one another at any given time. This approach is significant in applications such as robotics^25^, and ultimately suggests that there is an inertia individuals face in reacting to the current state of an epidemic. While a limitation of the simplified model is that it does not capture subtleties in disease transmission such as recovery times as well as classical models, we note that the model is sufficient to explain variation in the data and provides a rigorous data-driven approach to estimation. We believe such simplifying assumptions have value in epidemic modeling, though we hope in future work to provide a robust comparison to other, more complex mechanistic feedback models to determine the significance of this simplicity.

Our results indicate that the implicit feedback control policy used by individuals that best fits the observed data is that of a proportional-derivative (PD) control. That is, the magnitude of an individual’s reaction to the progress of the epidemic is proportional to both the observed number of cases and the weekly rate of change of cases. While the description of the PD control is implicit, we find that the parameters of the control correlate strongly with explicit control measures such as vaccination rates and mobility restrictions. As such, in future work we would like to further quantify and establish a causal link between explicit and implicit strategies for infectious disease mitigation. Ultimately, these initial results reinforce the validity of the closed-loop model and suggests possible mechanisms for improved policy design.

Specifically, we propose the modification of the control strategy to be a proportional-integral-derivative (PID) control, as opposed to a PD control. In such a strategy, the magnitude of an individual’s reaction would also depend on the cumulative number of cases so far. Such a modification could result in zero weekly COVID-19 cases, but the data suggests that no region is currently implementing a policy which has a statistically significant non-zero integral term in their COVID-19 response. In future work, we hope to assess the feasibility of an integral control in the context of infectious disease. Because augmenting the implicit control with a term proportional to the sum of previous case counts would eventually eradicate new cases, this suggests that policy makers should emphasize the cumulative costs of the pandemic when communicating with the public, even when current cases are low.

## Supplementary Information

Supplementary Information.

## Data Availability

Most data used in this article is publicly available, with the exception of SafeGraph mobility data. COVID-19 time series data is available at https://github.com/CSSEGISandData/COVID-19 for all regions considered in this work. For auxiliary data of explicit interventions, vaccination data is available at https://github.com/owid/covid-19-data and Google mobility data is retrieved from https://www.google.com/covid19/mobility/. Safegraph mobility data is available upon request at https://www.safegraph.com/academics.

## Code availability

Code needed to reproduce numerical results and figures for this work can be found at https://github.com/arnabsarker/implicit-control-epidemics, which includes the publicly available data used for the study.
