# Supplementary material for: Implicit feedback policies for COVID-19: why “zero-COVID” policies remain elusive
Source: Sci Rep. 2023 Feb 23;13:3173. doi: 10.1038/s41598-023-29542-8 (PMC9947911; doi:10.1038/s41598-023-29542-8)
Supplement: Supplementary file 1 — Supplementary Information. [file 41598_2023_29542_MOESM1_ESM.pdf]

Supplementary Information

A Proof of Proposition 1

Proof. First, note that for the two models to be equivalent, we must take the noise parameter in Eq. (2) to be a constant, i.e. $\sigma^2 = 0$. We then construct the parameterization as follows: For arbitrary but particular time series $\beta(t)$ and $\gamma(t)$, first define

$$\mathcal{R}^{SIR}(t) = 1 - \gamma(t) + \frac{\beta(t)}{N} S(t)\,.$$

Then, for

$$\mathcal{R}(0) = \mathcal{R}^{SIR}(0)\,, \quad \text{and} \quad \forall t \geq 1,\ \rho(t) = \frac{\mathcal{R}^{SIR}(t)}{\mathcal{R}^{SIR}(t-1)}\,, \quad ,$$

the model in Eq. (2) is equivalent to the time-varying SIR model in Eq. (3) . □

B Derivation of Eq. (5)

In this section, we show that Eq. (5) can be derived from the open-loop system Eq. (2) when the control takes the form Eq. (4) .

Rewriting Eq. (5) using the definition of $\mathbf{X}(t)$, we see that wish derive the following matrix equation:

$$\begin{bmatrix} \log I(t) \\ \log I(t+1) \\ \sum_{k=1}^{t} \log \rho(k) \end{bmatrix} = \begin{bmatrix} 0 & 1 & 0 \\ 0 & 1 & 1 \\ -\beta_2 & \beta_1 + \beta_2 & 1 \end{bmatrix} \begin{bmatrix} \log I(t-1) \\ \log I(t) \\ \sum_{k=1}^{t-1} \log \rho(k) \end{bmatrix} + \begin{bmatrix} 0 \\ \log \mathcal{R}(0) \\ -\beta_2 \log \mathcal{R}(0) + \beta_3 \end{bmatrix} + \boldsymbol{\eta}(t)\,,$$

where $\boldsymbol{\eta}(t)$ is a Gaussian random vector with mean 0 and variance σ^2.

We show this claim by showing that the three scalar equations associated with the matrix equations above hold. For the first equation, we note

$$\log I(t) = \log I(t)\,,$$

so clearly the top equation of the matrix multiplication holds, where the associated noise in the first entry of $\boldsymbol{\eta}(t)$ has mean and zero variance.

Second, we note that from Eq. (2) , by taking logarithms and and reindexing, we have:

$$\log I(t+1) = \log I(t) + \log \mathcal{R}(0) + \sum_{k=1}^{t-1} \log \rho(k) + \log \eta(t)\,.$$

Hence, the middle equation in the matrix equation also holds, since $\log \eta(t)$ is modeled as a Gaussian random variable.

Finally, the third matrix equation follows from the Eq. (4) , as

$$\begin{aligned} \sum_{k=1}^{t} \log \rho(t) &= \log \rho(t+1) + \sum_{k=1}^{t} \log \rho(k) \\ &= \beta_1 \times \log I(t) + \beta_2 \times (\log I(t) - \log I(t-1) - \log \mathcal{R}(0)) + \beta_3 + \sum_{k=1}^{t} \log \rho(k) \qquad \text{(Eq. (4))} \\ &= (\beta_1 + \beta_2) \times \log I(t) \ - \beta_2 \times \log I(t-1) + \sum_{k=1}^{t} \log \rho(k) - \beta_2 \times \log \mathcal{R}(0) + \beta_3\,. \end{aligned}$$

Here, again there is no noise associated with the equation so the model of $\boldsymbol{\eta}(t)$ can still be used.

Hence, we see that the closed loop equation Eq. (5) can be derived from the open-loop model Eq. (2) when the control input is set as Eq. (4) .

C Connection Between the Closed Loop Model and Time-Varying SIR Model

We note there is a connection between the SIR model in Eq. (3) and the closed loop model in Eq. (5) in the sense that Eq. (4) implies a particular structure for the time-varying parameters in Eq. (3) . Specifically, we see that from Eq. (4) ,

$$\rho(t) = I(t)^{\beta_1} \left(\frac{I(t)}{I(t-1)}\right)^{\beta_2} \mathcal{R}(0)^{-\beta_2} e^{\beta_3} \,. \tag{1}$$

Furthermore, from the above proof of Proposition 1, we see that the open-loop model and the time-varying SIR model are equivalent so long as

$$\rho(t) = \frac{N - N\gamma(t) + \beta(t)S(t)}{N - N\gamma(t-1) + \beta(t-1)S(t-1)} \,. \tag{2}$$

Equating these two representations of $\rho(t)$, we find that if $\gamma(t)$ and $\beta(t)$ obey

$$\left(\frac{\gamma(t)}{\gamma(t-1)}\right) \left(\frac{\frac{N+\beta(t)S(t)}{\gamma(t)} - N}{\frac{N+\beta(t-1)S(t-1)}{\gamma(t-1)} - N}\right) = I(t)^{\beta_1} \left(\frac{I(t)}{I(t-1)}\right)^{\beta_2} \mathcal{R}(0)^{-\beta_2} e^{\beta_3} \,, \tag{3}$$

which can be done by equating terms of the product with one another, then the two models can be equivalent. For example, if for all $t \geq 1$,

$$\frac{\gamma(t)}{\gamma(t-1)} = I(t)^{\beta_1} e^{\beta_3} \,, \quad \text{and} \quad \frac{\frac{N-\beta(t)S(t)}{\gamma(t)} - N}{\frac{N-\beta(t-1)S(t-1)}{\gamma(t-1)} - N} = \left(\frac{I(t)}{I(t-1)}\right)^{\beta_2} \mathcal{R}(0)^{-\beta_2} \,, \tag{4}$$

then we see a specific choice of $\beta(t)$ and $\gamma(t)$ can be imposed by the closed loop model in Eq. (5) . Specifically, this particular choice would result in

$$\gamma(t) = \left(\prod_{k=1}^{t-1} I(k)\right)^{\beta_1} e^{t\beta_3}\gamma(0) \,,$$

and

$$\beta(t) = \left(N - \gamma(t)\left(\left(\frac{I(t)}{I(t-1)}\right)^{\beta_2} \mathcal{R}(0)^{-\beta_2} \left(\frac{N - \beta(t-1)S(t-1)}{\gamma(t-1)} - N\right) + N\right)\right) / S(t) \,.$$

That is, one possible interpretation of the implicit feedback policy is that the magnitude of $\gamma(t)$ increases as the cumulative product of cases increases, and that $\beta(t)$ is inversely proportional to the remaining susceptible population and is a complex function of recovery rates $\gamma(t)$ and the rate of change of cases $I(t)/I(t-1)$.

D Additional Figures

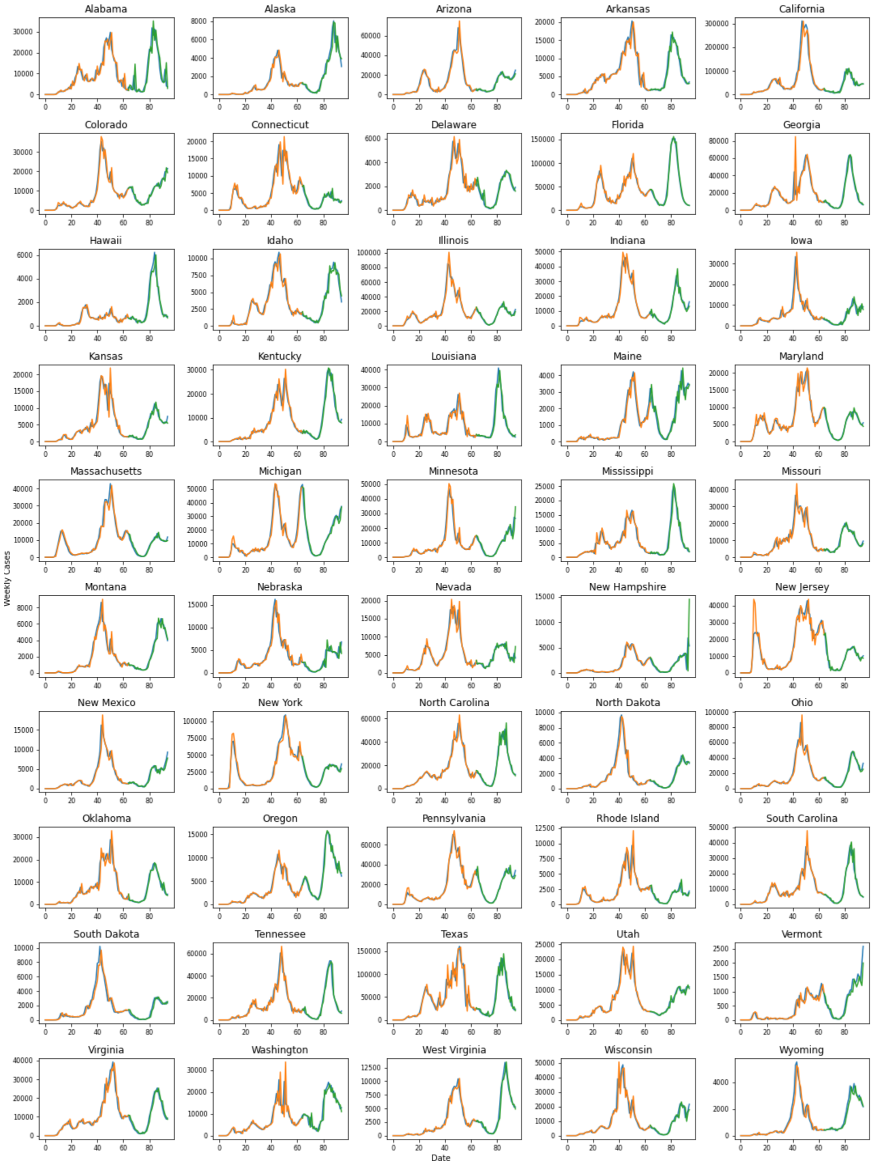

Figure S1: Case counts of US states as modeled by the dynamical system. One week ahead predictions using this method are shown in orange (in sample) and green (out of sample), and the true data is shown in blue. We find that using data from roughly the first year of the pandemic results in surprisingly accurate performance on later data.

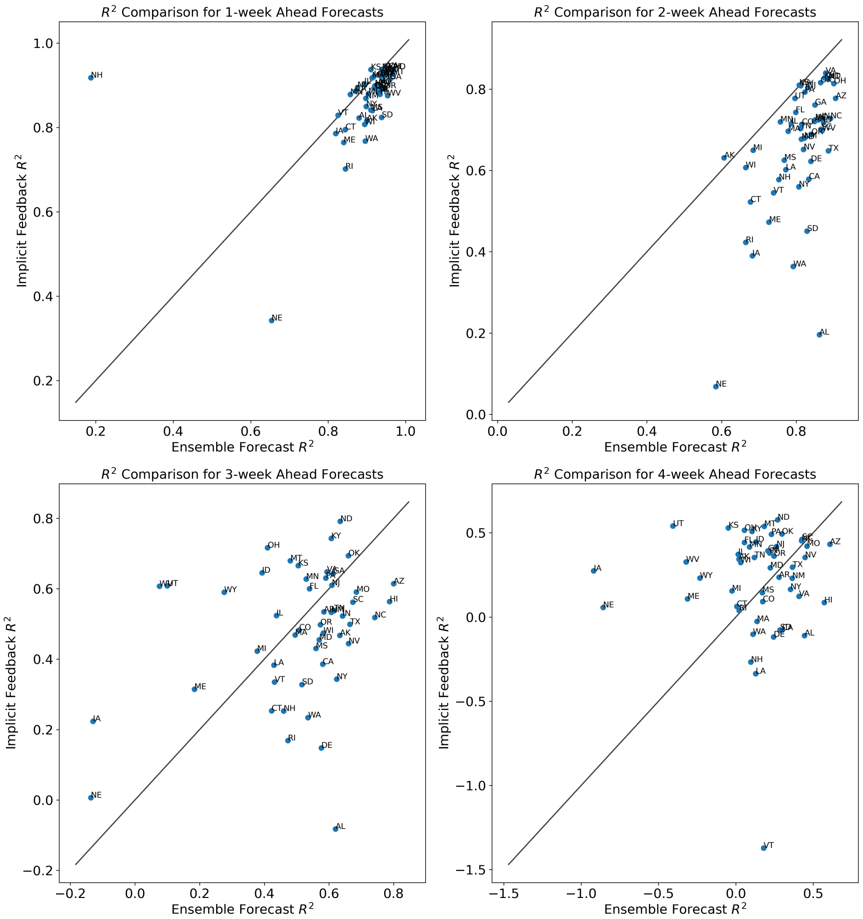

Figure S2: k-week ahead forecasts for the implicit control approach of Eq. (5) compared to ensemble forecasts [1], across the 50 United States. Implicit feedback parameters are learned using data up to each time t, and then forecasts are computed to create a prediction for each time $t + k$ for k between 1 and 4. We find that the implicit feedback approach has comparable R^2 scores to the ensemble approach for $k = 1, 3, 4$, as the R^2 scores can not be distinguished by a t-test. Moreover, we find that the implicit feedback approach outperforms ensemble predictions on 21 and 30 states for 3 and 4 week ahead forecasts, respectively, suggesting that as the forecast target becomes further from the forecasting date, the method improves, consistent with previous results on using feedback in forecasting [2].

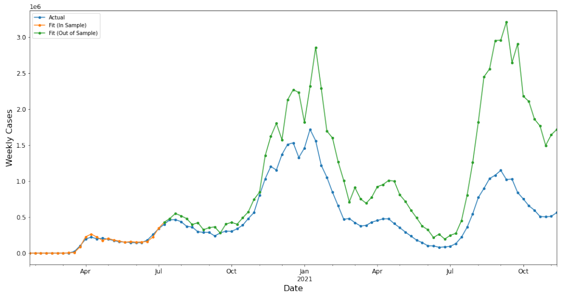

(a) PID controller learned with data from the first wave of the COVID-19 pandemic in the United States.

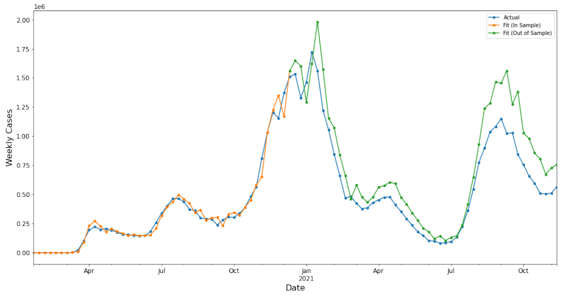

(b) PID controller learned with data from the first two waves of the COVID-19 pandemic in the United States.

Figure S3: Implementation of a PID controller learned using the single state $X(t) = \log I(t)$ (i.e., $X_2(t)$ in Eq. (5)). The PID controller has the form $u(t) = k_P X(t) + k_I \sum_{\tau=1}^{t} X(\tau) + k_D(X(t) - X(t-1))$, and we assume the system has the form $X(t+1) = X(t) + u(t)$. Learning for the first two waves results in an out of sample R^2 value of 0.085, and from the first three waves results in $R^2 = 0.81$, compared to an R^2 of 0.905 using the model in Eq. (5) trained on the first two waves of data, implying that the PID method requires more data to provide a worse explanation out of sample. This suggests the model in Eq. (5) provides a better fit to the data.

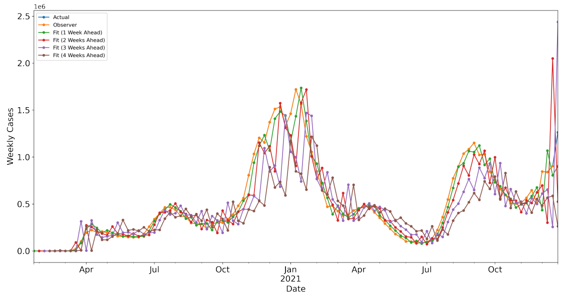

(a) Predictions using statistical estimates.

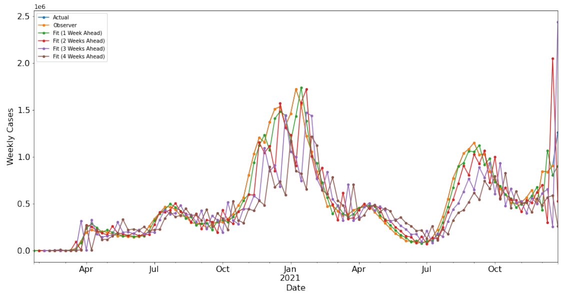

(b) Predictions using estimates from an observer system.

Figure S4: k-week ahead forecasts using the system in Eq. (5) using statistical estimates (a) or an observer model (b) which uses an estimate of $X_1(t)$. Both models are learned with a fixed training period using the first 35 weeks of data. For statistical estimates, future values of $X_1(t)$ are estimated through feeding back estimates into Eq. (5) , and for the observer system a parallel system is used. We find that using the observer system results in the same performance compared to the statistical method, and that with the fixed training window the closed loop method works best in the short term for 1 to 2 week ahead predictions.

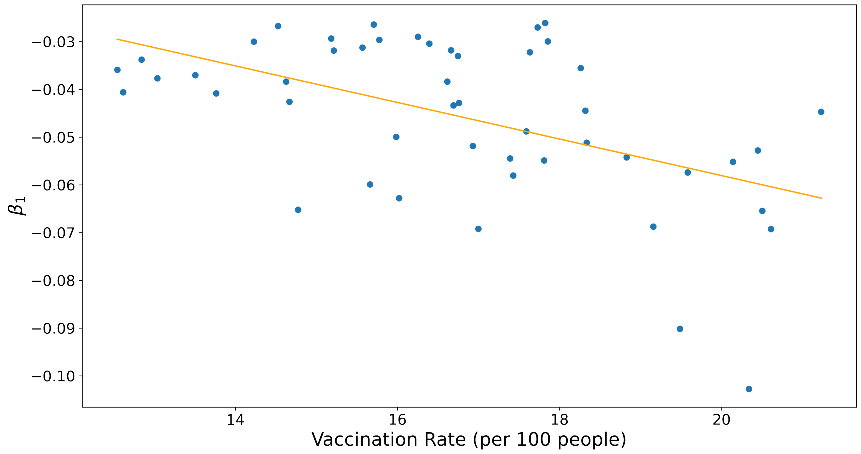

Figure S5: Comparison of learned β_1 parameters in each state to observed vaccination rates from https://github.com/owid/covid-19-data, where each point represents a state and the orange line is the line of best fit. We see that higher vaccination rates correspond to larger magnitudes of the β_1 parameter.

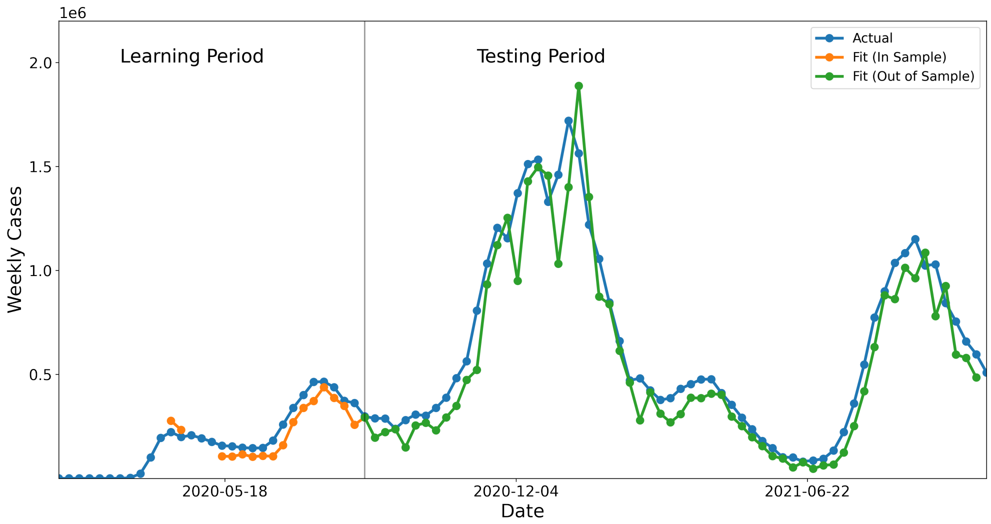

Figure S6: Forecasts of United States COVID-19 Cases using data available at each date. Because case data is adjusted after the fact, we find that using data available at each date results in a small deterioration in prediction performance, with an out of sample R^2 score of 0.901 as opposed to 0.948 using data as of March 2022.

State	R^2 of Implicit Feedback Predictions	R^2 of CDC Forecasts	R^2 of SEIR Model
Alabama	0.761268	0.870218	-7.466713
Alaska	0.895081	0.860655	-6.097458
Arizona	0.948373	0.912740	-16.846574
Arkansas	0.919352	0.904562	-5.102133
California	0.846362	0.815874	-38.857268
Colorado	0.912670	0.928491	-17.163285
Connecticut	0.787693	0.770680	-13.130171
Delaware	0.901341	0.960540	-23.524277
Florida	0.984187	0.934497	-16.086748
Georgia	0.967953	0.951781	-29.483336
Hawaii	0.910708	0.919715	-3.725094
Idaho	0.936136	0.919588	-86.226929
Illinois	0.923297	0.902908	-25.749134
Indiana	0.902190	0.925885	-61.444810
Iowa	0.813681	0.777314	-8.914433
Kansas	0.930767	0.851023	-8.554606
Kentucky	0.961193	0.927413	-4.442309
Louisiana	0.895427	0.894736	-25.563110
Maine	0.854193	0.871618	-13.050083
Maryland	0.941903	0.949353	-29.439844
Massachusetts	0.920278	0.905620	-12.569417
Michigan	0.937189	0.953322	-20.089249
Minnesota	0.876778	0.852998	-8.951495
Mississippi	0.931766	0.887371	-5.910868
Missouri	0.952072	0.903154	-12.156490
Montana	0.956009	0.956591	-3.621835
Nebraska	0.648975	0.606238	-16.876655
Nevada	0.786819	0.794385	-32.207349
New Hampshire	0.337766	0.012839	-32.442966
New Jersey	0.913044	0.947180	-7.806754
New Mexico	0.918922	0.858679	-12.438151
New York	0.948178	0.925340	-8.925778
North Carolina	0.892444	0.908425	-7.784810
North Dakota	0.969735	0.964937	-3.343721
Ohio	0.965163	0.950134	-19.912066
Oklahoma	0.957803	0.922573	-7.961322
Oregon	0.949682	0.935502	-2.391805
Pennsylvania	0.937376	0.950664	-20.954253
Rhode Island	0.756697	0.821439	-17.282322
South Carolina	0.905781	0.915286	-9.553737
South Dakota	0.929809	0.936414	-13.868844
Tennessee	0.935708	0.920788	-41.321343
Texas	0.884807	0.915912	-10.169516
Utah	0.945423	0.865068	-16.726585
Vermont	0.832917	0.819457	-45.831707
Virginia	0.956532	0.954946	-16.765430
Washington	0.847929	0.893503	-5.760364
West Virginia	0.957164	0.947691	-2.813714
Wisconsin	0.897933	0.870112	-12.464855
Wyoming	0.924037	0.919302	-9.180029
**Mean**	0.891370	0.877309	

Table S1: r^2 metric of 1 week ahead forecasts using the implict feedback approach summarized in Eq. (5) compared to the r^2 scores of 1 week ahead forecasts from a state of the art ensemble model [1] and a simple SEIR model [3].

Dependent Variable	Independent Variable	Coefficient Value	p-value	Null Hypothesis Rejected
β_2	Mobility Component 2	0.006	< 0.001	✓
β_2	Natural Immunity (%)	16.852	< 0.001	✓
β_2	Mobility Component 1	0.002	< 0.001	✓
β_1	Mobility Component 2	0.001	0.001	✓
β_1	Natural Immunity (%)	5.233	0.001	✓
β_1	Mobility Component 1	0.000	0.443	

Table S2: Hypothesis tests comparing different policies to learned β_1 and β_2 values across both US states and countries around the globe. Across heterogeneous regions, we find that β_1 and β_2 tend to correlate significantly with observed measures such as mobility and the level of natural immunity. Hypotheses are rejected at the $\alpha = 0.05$ level using the Bonferroni Correction.

Dependent Variable	Independent Variable	Coefficient Value	p-value	Null Hypothesis Rejected
β_1	Vaccination Rate (%)	-0.004	< 0.001	✓
β_1	Mobility Component 2	-0.000	0.004	✓
β_1	Mean Distance Traveled From Home	-0.028	0.007	✓
β_2	Median Percentage Time Home	0.016	0.018	✓
β_2	Mean Distance Traveled From Home	-0.210	0.044	
β_2	Mobility Component 1	-0.001	0.056	
β_1	Median Time Spent Away From Home	0.000	0.064	
β_2	Vaccination Rate (%)	-0.019	0.073	
β_1	Mask Mandate	-0.017	0.079	
β_2	Natural Immunity (%)	7.116	0.128	
β_2	Mobility Component 2	0.002	0.184	
β_1	Natural Immunity (%)	0.608	0.210	
β_2	Mask Mandate	-0.057	0.550	
β_2	Median Time Spent Away From Home	-0.001	0.582	
β_1	Median Percentage Time Home	-0.000	0.853	
β_1	Mobility Component 1	-0.000	0.962	

Table S3: Hypothesis tests comparing different policies to learned β_1 and β_2 values in the United States only. We find that β_1 correlates with vaccination rates in each state as well as mobility, and that β_2 correlates with mobility metrics. Here, vaccination data is taken from https://github.com/owid/covid-19-data and mobility auxiliary mobility data is taken from SafeGraph ("Median Time Spent Away From Home," "Mean Distance Traveled From Home," , "Median Percentage Time Home") and Google (Mobility Components 1 and 2, which are principal components of the 6 mobility metrics provided by Google). Hypotheses are rejected at the $\alpha = 0.05$ level using the Bonferroni Correction.

References

[1] Evan L Ray, Nutcha Wattanachit, Jarad Niemi, Abdul Hannan Kanji, Katie House, Estee Y Cramer, Johannes Bracher, Andrew Zheng, Teresa K Yamana, Xinyue Xiong, et al. Ensemble forecasts of coronavirus disease 2019 (covid-19) in the us. Cold Spring Harbor Laboratory Press, 2020.

[2] Hazhir Rahmandad, Ran Xu, and Navid Ghaffarzadegan. Enhancing long-term forecasting: Learning from covid-19 models. *Available at SSRN 3906690*, 2021.

[3] Herbert W Hethcote. The mathematics of infectious diseases. *SIAM review*, 42(4):599–653, 2000.
